# 3D Printing and The Evolution Of Partial Hand Prostheses: My Journey from Theory To Practice

**DOI:** 10.33137/cpoj.v6i2.42139

**Published:** 2023-12-22

**Authors:** C.M Baschuk

**Affiliations:** Point Designs, LLC, Bountiful, UT, USA.

**Keywords:** Prosthetics, Additive Manufacturing, 3D Printing, Partial Hand, Finger Amputation, Material Science, CAD, Biomechanics, User-Centered Design, Rehabilitation, Multi-jet

## Abstract

The world of prosthetics has been undergoing significant changes, with the evolution of materials, design techniques, and manufacturing methodologies converging to redefine the landscape. Central to this narrative is the imperative for a holistic approach, harmonizing the trinity of materials, design, and methodologies to yield optimal outcomes. This balance is especially pivotal for the overlooked yet significant segment of those with partial hand and finger differences. Historically, this demographic has been underserved, with rehabilitation and prosthetic innovations often falling short. The sheer prevalence of partial hand differences underscores the urgency of tailored solutions. Traditional fabrication methods like wet lamination have posed challenges, particularly in aligning and efficiency. The advent of additive manufacturing has been transformative. The case of designing and printing a partial finger socket for Point Designs, LLC's Point Partial finger highlights this paradigm shift. Where conventional techniques demanded hours, digital design and 3D printing have condensed the process to mere minutes, without compromising on quality. This is not merely a win in terms of time efficiency; the implications for the end users are profound, ensuring a more customized and efficient solution. The journey underscores the potential of blending technology and traditional prosthetic knowledge, pointing towards a future where prosthetics align more seamlessly with users' needs.

## INTRODUCTION

Additive manufacturing (AM), commonly known as 3D printing, is revolutionizing the field of prosthetics, particularly in the space of partial hand and/or finger prostheses. Unlike traditional methods which often require molds, casts, and extensive manual labor, additive manufacturing provides a more streamlined, customizable, and cost-effective approach.^1^ There are three distinct areas in which additive manufacturing offers additional advantages over traditional prosthetic fabrication processes such as laminations and vacuum forming (**Table 1**).

**Table 1:** Benefits of 3D Printing in Prosthetic Design and Fabrication.

Category	Benefits
Materials	Expansive array of materials, from flexible thermoplastics to strong polymers. Ability to layer or combine materials for desired textures, flexibility, and strengths. Nuanced material choices tailored to user needs.
Design Flexibility	Precise tailoring to the user's anatomy using digital software. Incorporation of advanced features like lattice structures for weight reduction. User-driven designs with aesthetic and functional preferences.
Manufacturing Methods	Customizability inherent in layer-by-layer additive manufacturing. Ability to create complex structures unachievable with traditional methods. Precise control over prosthetic socket wall characteristics. Digital simulation and validation prior to physical production. Ease of making adjustments to designs.

The success of a prosthesis hinges on the proper integration of materials, design, and manufacturing methods by the prosthetist. When balanced, these components yield a prosthesis that harmoniously merges technology with the user's daily life. This interdependence is of particular importance in the provision of partial hand and finger prostheses.

## UPPER LIMB DIFFERENCES

The partial hand and/or finger difference community is a significant yet often overlooked segment of the broader limb difference population. Despite being the largest group of individuals with upper limb differences, this demographic has historically been underserved in terms of research attention, rehabilitation resources, and prosthetic innovation. They are often told their loss or difference is only minor and that they will just adapt to it. A study by Ziegler-Graham et al. indicates that partial hand amputations account for nearly 90% of all upper limb amputations, underscoring the prevalence of this specific condition.^2^ However, prosthetic solutions and rehabilitation programs have disproportionately focused on more proximal levels of amputation, such as transradial or transhumeral, leaving a gap in care for those with partial hand amputations.^3^

Historically, prosthetic options for partial hand and finger differences were limited in scope and functionality in large part due to the difficulty of integrating the prosthesis with the residual anatomy. Early solutions were primarily cosmetic, offering passive silicone or rubber prostheses that aimed to replicate the appearance of missing fingers or parts of the hand without providing any meaningful function.^4^ In the 1970's, advances in materials and technology introduced mechanical finger and partial hand prostheses. These devices, made of materials like stainless steel, aluminum, and durable plastics, provided some degree of grip or pinch through cable-operated or body-powered mechanisms.^5^ While they marked an improvement over cosmetic options, they still lacked the dexterity and intuitiveness of natural finger movement.^6^ The innovation in this space stagnated until the early 2000's.

The challenges a prosthetist faces in meeting specific needs of individuals with partial hand and/or finger differences are complicated by the fact that no two hand presentations are ever exactly the same. The underlying cause of the partial hand or finger difference combined with differing surgical paradigms and general lack of knowledge amongst surgeons regarding prosthetic options for this population creates a wide variety of presentations even amongst individuals with the same parts of their hands or fingers involved.^7^ Meeting the functional needs of this patient population has been quite challenging. Many surgeons don't even refer these individuals to prosthetists. When they do, the local prosthetist may not be aware of all the options currently available to restore the form and function of the involved hand and/or fingers through prosthetic rehabilitation.

The intricacies and functional demands of hands, coupled with the unique biomechanics of fingers, require specialized knowledge and design considerations. Yet, due to their foundational experience in lower extremity socket designs and lack of educational resources on the topic, many prosthetists unconsciously gravitate towards utilizing familiar techniques. This results in partial hand prosthetic sockets that might be shaped like a hand, but from a design, materials, and manufacturing methods standpoint are essentially lower limb prosthetic sockets fit on hands. Such designs, while made with the best intentions, may not capture the full range of motion, tactile sensitivity, and versatility required of a hand prosthesis. Hence, there's a pressing need for specialized training and awareness among prosthetists to ensure that upper limb prostheses, particularly for the hand, are tailored for optimal function and user comfort. Given these challenges, would the more widespread adoption of additive manufacturing in the production of partial hand prostheses be beneficial?

## 3D PRINTING

If I had to give you a one-word answer now it would be an emphatic, “Yes!” But that hasn't always been the case. In fact, I had significant reservations about the way in which it was being applied for several years starting around 2014. The idea of using 3D printing for prosthetics began gaining traction in the early 2010s, particularly with the advent of more accessible and affordable desktop 3D printers utilizing fused deposition modeling (FDM). One of the early and most notable 3D-printed prosthetic projects was the Robohand, which was a partial hand prosthesis co-developed by Richard Van As, a carpenter from South Africa who lost several of his fingers in a woodworking accident, and Ivan Owen, a special effects artist and puppeteer from the United States.^8,9^

Their efforts generated a lot of hype over the use of additive manufacturing and its application to upper limb prostheses. Some groups tried to emphasize the lower cost of materials, sans the clinical care needed to effectively provision upper limb prostheses, to vilify our profession; making prosthetists out to be greedy for charging upwards of $80,000 for a myoelectric prosthesis when a prosthesis could be printed at home for only $50. In 2015, Marvel Studios and Disney even got involved when they had Robert Downey Jr, as Ironman, deliver a 3D printed prosthesis to a young boy that was made to look like the Ironman gauntlet.^10^ There was no indication that a prosthetist was involved in the fitting process even though it occurred in a US state that has licensure for prosthetics and orthotics. The social and traditional media loved the story, and it went viral.

This created a stir within the national prosthetic and orthotic organizations in the United States. Some went so far as to call for an outright ban on the use of 3D printing in prosthetics and orthotics. The problem was those calling for bans were erroneously vilifying the manufacturing method, just like those 3D printing groups were erroneously vilifying us, the manufacturers. Concerned about these misconceptions, I wrote an article promoting the acceptance and collaboration around 3D printing in prosthetics.^11^ I saw the potential that additive manufacturing could have for upper limb prosthetics and feared that due to misconceptions and unsafe applications it would be cast off by our profession. One of additive manufacturing's greatest strengths is that it can produce high complexity, low-volume components in a cost-efficient manner. Upper limb prostheses are exactly that, highly complex and produced in low volumes. However, they also need to be durable, and the FDM manufacturing methods being used were lacking in this area.

A real breakthrough in the practical application of additive manufacturing in prosthetics came with the increased accessibility of powder bed printing technologies such as selective laser sintering (SLS) and, in 2016, multi-jet fusion (MJF). The introduction of materials like PA12 and PA11, which are nylon variants, ushered in a new era of prosthetics. These methods were able to produce end products with greater strength, homogeneity, and higher resolution, than FDM. They also lacked the distinct layer lines of FDM. These advancements allowed for the creation of innovative prosthetic designs, previously inconceivable with traditional methods, marking a revolutionary shift in the prosthetic manufacturing domain.

The arrival of the MJF technology influenced my decision to begin implementing a hybrid workflow into my clinical practice. Between 2019 and 2021 I began to utilize hand casting for shape capture in order to achieve an optimal impression to start from. Then I would digitize the impression with a 3D scanner and utilize Geomagic Freeform Plus software to rectify the model and to design the prosthesis. I initially found myself recreating designs of prostheses that I could have traditionally fabricated. As I became more comfortable with the principles of Designing for Additive Manufacturing (DfAM) I would begin to integrate more complex geometries and features; creating prostheses that couldn't have been produced through any other method than additive manufacturing.

I would generally use FDM prints for my diagnostic sockets and then multi-jet fusion printing for the definitive sockets. I discovered that the sockets I was making were lighter and lower profile because of the ability to fine tune the socket and wall geometry with a freedom that I hadn't experienced before. Not to mention, my favorite feature of digital socket fabrication, the undo button; a luxury not found in plaster modifications or laminations.

In 2021, I embarked on a transformative journey by designing and printing my first partial finger socket for Point Designs, LLC's Point Partial finger. My previous reliance on traditional wet lamination methods proved cumbersome and inefficient, often grappling with the small size and alignment complexities inherent in such a design. An alignment mishap in a previous project further highlighted the limitations of these traditional techniques, sparking my interest in the potential of additive manufacturing.

Collaborating with Point Designs, we innovated a mounting bracket that seamlessly melded with additively manufactured sockets. Where traditional methods consumed several hours, my foray into digital design and printing shrunk the fabrication process of the Point Partial socket to just 30 minutes of design time. Printing took a passive role in the background, allowing me to focus on other responsibilities. Upon receipt from the printer, assembling the components was a matter of minutes. From several laborious hours, the process was now streamlined to roughly 40 minutes – a testament to the game-changing nature of additive manufacturing.

What I initially saw as a time-saving measure for myself had broader implications. This advancement was not just about clinician convenience, but it signified a paradigm shift with profound benefits for the end users, enhancing their experience and changing the prosthetic landscape.

In late 2022 utilizing the principles of DfAM, I came up with an idea for a radically different design of a partial hand prosthesis. It was designed to be rigid where the prosthetic fingers mounted, but also minimized the area of the rigid portions and replaced them with flexible regions where the residual hand needed to move. The first person that I fit with this design was Jeff Soelberg.

**Figure 1:**
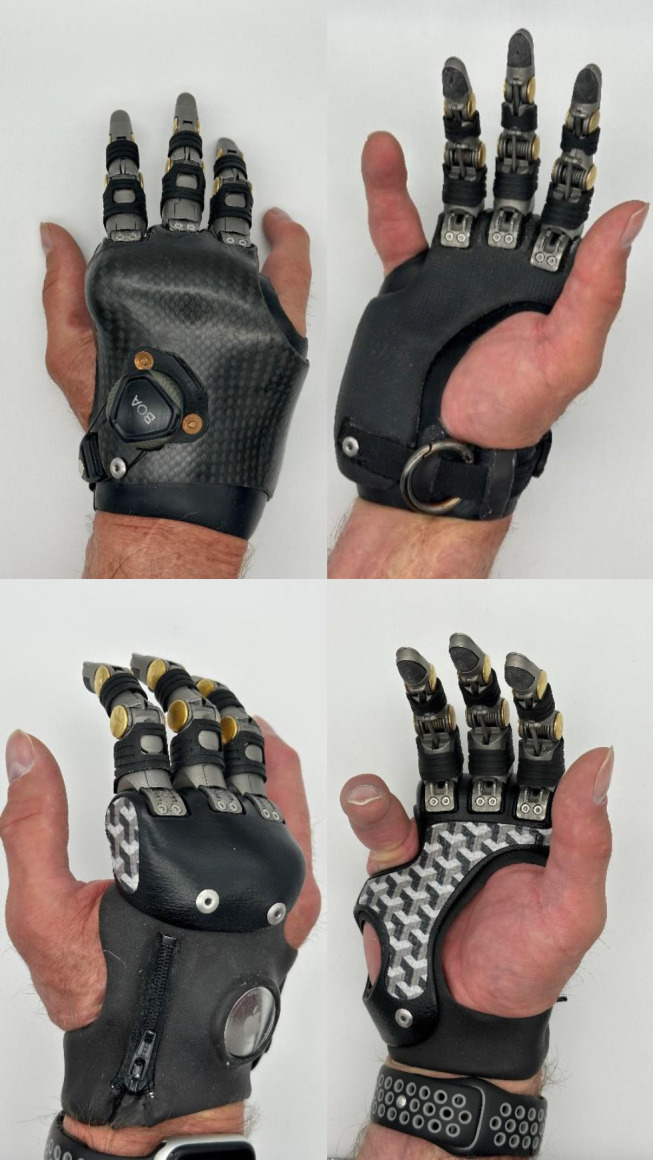
Top row showing dorsal and palmar views of Jeff's carbon fiber laminated prosthesis. Bottom row showing dorsal and palmar views of Jeff's additively manufactured prosthesis. The significant difference in bulk and rigid areas between the two prostheses is clearly seen.

After an industrial accident in 2016, Jeff lost digits 2-4 on his right hand. Over the years, he tried multiple prostheses; three of them having carbon fiber laminated frames. Each of the carbon fiber sockets felt different to Jeff. Collaborating with Point Designs in January 2023, Jeff tried the customized 3D printed prosthesis for the first time. He expressed, “*The difference between a carbon fiber laminated and the 3-D printed frame is night and day*.” Jeff instantly felt that the 3D printed frame would be transformative. Previously, he wore his prosthesis sporadically depending on his activities. Since acquiring the 3D printed prosthesis, he puts it on in the morning and wears it until bedtime, describing the fit as a “well-tailored glove”. (**Figure 1**) One of his major challenges had been finding suitable work gloves. Earlier, he always had to customize gloves to wear them. With the 3D printed design, he can now easily purchase gloves off-the-shelf. He mentioned, “*Before my prosthetic was a tool. Today, it's an extension of my right hand. I feel better with it on than not having it on.*”

His experience with the new design has transformed his daily life. He confidently recommends the 3D printed frame, highlighting its reproducibility. He emphasized, “If something happens, you just call your CPO, and they print a new one that fits like the old one. It's much easier than the old way.” With such strong endorsement and the evident benefits of flexibility, customization, and the PA12 nylon material, Jeff's journey showcases the promise and impact of advancements in prosthetic technology. He firmly states, “*I never want to go back to a carbon laminated prosthesis.*” I've found numerous advantages of additive manufacturing over traditional fabrication methods in prosthetic design. While traditional methods require manual adjustments and often result in imperfect fits, 3D printing allows for precise, digital planning that ensures components fit seamlessly on the first try.

This reduces the painstaking manual work, like drilling and filing, previously needed to achieve a flush finish on prostheses. Additive manufacturing also boasts reproducibility; if a prosthesis breaks, is lost, or stolen, the design can be easily re-evaluated, adjusted if needed, and reprinted at a minimal cost, ensuring the initial hard work in optimizing the fit is never wasted.

## CONCLUSION

In conclusion, additive manufacturing is not just a new tool in the prosthetist's arsenal; it's a paradigm shift. It democratizes the design and production process, making advanced, customized partial hand prostheses more accessible and adaptable to the end user. There is a misconception by some that 3D printing is a panacea for prosthetic rehabilitation. Yet, there are still others for whom 3D printing is anathema. The appropriate application of 3D printing in this space is one of the biggest challenges that is still being debated. It makes no sense to design a prosthesis according to the same designs we have historically made and then 3D print it. If you can make the prosthesis with traditional fabrication methods effectively, why would you go through the trouble of 3D printing it? There is so much more that can be done and integrated into a prosthesis with a shift in the ethos to a DfAM paradigm, than can be done with traditional manufacturing. The advancements seen in the last 10 years with prosthetic options available for individuals with partial hand and/or finger differences as a result of the appropriate application of additive manufacturing materials, methods, and designs is a clarion call for further innovation in this space.

Of all the places where additive manufacturing has been applied in prosthetic rehabilitation, the application to prostheses for the partial hand and/or finger difference populations has been one of the most if not the most transformative and will continue to be going forward. Afterall, 3D printed partial hand and finger prostheses were the catalyst that brought visibility and growing widespread application of additive manufacturing into prosthetic rehabilitation.

## CALL TO ACTION

You don't need to invest a lot of money or buy a 3D printer of your own to begin the process of implementing additive manufacturing into your own clinical practice. I would encourage anyone who wants to get started to download Meshmixer, a free organic modeling software and spend some time on Youtube watching tutorials. That is what I did initially, and it doesn't cost anything but your time. Learning the principles of DfAM takes some time to comprehend but is essential to produce functional prostheses. Finally, I would encourage you to reach out to individuals on LinkedIn, for example, that you see posting about the prostheses that they are creating with additive manufacturing. Many of them, including myself, are more than willing to become a mentor to you.

## DECLARATION OF CONFLICTING INTERESTS

I currently am employed by Point Designs, LLC as the Director of Clinical Services and have been since May of 2022. This work is my own from my own personal experience both prior to my employment with Point Designs as well as during. All opinions or statements are my own and do not necessarily reflect the views or opinions of Point Designs, LLC.

## SOURCES OF SUPPORT

None.

## AUTHOR SCIENTIFIC BIOGRAPHY

**Figure:**
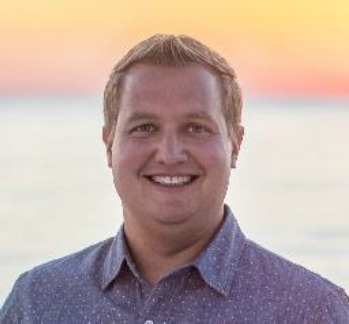


**Chris Baschuk,** MPO, CPO, FAAOP(D), stands at the forefront of upper limb prosthetic rehabilitation, fervently advocating for the transformative potential of additive manufacturing. As the Director of Clinical Services at Point Designs, LLC, he has pioneered the integration of advanced manufacturing techniques with silicone customization to enhance prosthetic solutions for individuals with partial hand and finger differences. A graduate of the University of Utah in Biomedical Engineering and UT Southwestern Medical Center in Prosthetics and Orthotics, Chris's academic and research contributions have been profound. Recognized as a Fellow with Distinction by the American Academy of Orthotists and Prosthetists, he has chaired the Upper Limb Prosthetics Society since 2017. Chris's extensive peer-reviewed publications and global lectures underscore his influence in the field. Beyond academia, he passionately advocates for prosthetic rehabilitation access, ensuring optimal care for those in need.
